# Physiological and Metabolic Changes of Purslane *(Portulaca oleracea* L.) in Response to Drought, Heat, and Combined Stresses

**DOI:** 10.3389/fpls.2015.01123

**Published:** 2016-01-07

**Authors:** Rui Jin, Yanping Wang, Ruijie Liu, Junbo Gou, Zhulong Chan

**Affiliations:** ^1^Key Laboratory of Plant Germplasm Enhancement and Specialty Agriculture, Wuhan Botanical Garden, Chinese Academy of SciencesWuhan, China; ^2^University of Chinese Academy of SciencesBeijing, China

**Keywords:** purslane, individual stress, combination of drought and heat, physiological analysis, metabolites

## Abstract

Purslane (*Portulaca oleracea* L.) is a fleshy herbaceous plant. So far, little information is available on the response of this plant to combined drought and heat stress. In this study, changes in physiological and metabolic levels were characterized after treatments with drought, heat and combined stresses. Both individual and combined stress treatments increased malondialdehyde (MDA), electrolyte leakage (EL), ${\text{O}}_{2}^{•-}$ and activities of superoxide dismutase (SOD), peroxidase (POD), while declined chlorophyll content. No significant differences were found between control and treatments in leaf water content (LWC) and catalase (CAT) activity. Additionally, 37 metabolic compounds were detected in purslane. Through pathway analysis, 17 metabolites were directly involved in the glycolysis metabolic pathway. The present study indicated that combined drought and heat stress caused more serious damage in purslane than individual stress. To survive, purslane has a high capability to cope with environmental stress conditions through activation of physiological and metabolic pathways.

## Introduction

During the evolution, plants have developed complex strategies to ensure their survival and reproduction especially under adverse environment conditions (Barnabas et al., 2008). With the global climate change, plants currently encounter harsh environments and are exposed to different stress factors in combination more often. Drought and heat are two representative abiotic stresses that usually appear in the field simultaneously and are the primary environmental stresses that determine the distribution and productivity of plants in tropical and subtropical areas (Mittler et al., 2001; Rizhsky et al., 2002). Plants respond to heat and drought by a progressive adjustment at physiological status and metabolic level with sustained and transient metabolic alterations. Previous studies showed that, drought or heat causes oxidative stress because of production of reactive oxygen species (ROS), including hydrogen peroxide. ROS accumulation leads to lipid peroxidation and thereby causes the damage of cell membrane stability, photosynthetic apparatus and chlorophyll biosynthesis (Smirnoff, 1993). As a result, oxidative damage of cellular components, inhibition of photosynthesis, dysfunction of metabolism, and damage of cellular structures lead to growth perturbation, reduced fertility, and premature senescence (Qin et al., 2007; Krasensky and Jonak, 2012).

Plants respond to multiple stresses differently from how they do to individual stress (Atkinson and Urwin, 2012). The evidence showed that the combination of heat and drought has more obvious detrimental effect on the growth and productivity of some crops and grasses compared with individual drought or heat stress (Savin and Nicolas, 1996). The molecular and metabolic responses of plants to the combination of two stresses are different and cannot be speculated from the response of plants subject to individual stress (Rizhsky et al., 2004; Mittler, 2006; Suzuki et al., 2014). Different stresses perhaps induce antagonistic or synergetic responses. For example, plants open their stomata to decrease the temperature of the leaves by transpiration when subjected to heat, but plants are unable to open their stomata when subjected to drought and heat simultaneously for avoiding unnecessary water loss. As a result, leaf temperature remains high (Rizhsky et al., 2002; Barnabas et al., 2008). Drought and heat imposed simultaneously might influence signals that control gas exchange (Prasch and Sonnewald, 2013). In addition, a combination of drought and heat was found to alter plant metabolism in a unique way in contrast to the effect of different stresses alone (Jiang and Huang, 2001; Rizhsky et al., 2004). Xu and Zhou (2006) found that high temperature, combined with severe soil drought might damage the function of PSII, weaken nitrogen anabolism, accelerate protein catabolism and provoke lipid peroxidation in perennial grass.

Purslane *(Portulaca oleracea* L.), an annual herb with succulent leaves may grow prostrate or erect depending on light availability (Chauhan and Johnson, 2009), which is distributed all over the world, and grows well in diverse geographical environments (Lee et al., 2011; D'Andrea et al., 2014). Purslane belongs to family Portulacaceae and is classified as C_4_ plant, which is listed as one of the most useful medicinal plants and named “Global Panacea” by World Health Organization (Sultana and Rahman, 2013). It is rich in antioxidant vitamins and omega-3 fatty acids (Rahdari et al., 2012) and can be used as a vegetable as well as for various curative purposes in health care especially in preventing some cardiovascular diseases and maintaining a healthy immune system (Simopoulos, 2004; Uddin et al., 2014). Rahdari and Hoseini (2012) reported the effect of drought on germination, proline, sugar, lipid, protein, and chlorophyll content variations in purslane. The results indicated that purslane was tolerant to drought stress and could be a promising candidate to be used in ecosystem restoration in arid and semi-arid regions. Yang et al. (2012) investigated the mechanisms underlying purslane's tolerance to high temperature and high humidity stresses through physiological and proteomic approaches, and the results suggested that purslane deployed the multiple strategies to cope with combined stresses. Multi-strategies, from induction of several metabolites to the transitory development of a CAM-like metabolism, were involved in enhancing adaptation to drought stress in purslane (D'Andrea et al., 2014).

Previously we reported physiological changes of purslane after progressive drought stress and rehydration treatment and the results indicated that purslane could recover from drought induced damages after 24 h rehydration (Jin et al., 2015). However, the effect of the combination of heat and drought on purslane is largely unknown. In this study, to fully understand how plants sense combined stress, we presented a new perspective in physiological and metabolic responses of purslane to individual drought, heat and combined drought and heat stresses. The results indicated that combined stress imposed more severe damage on purslane than drought or heat stress alone.

## Materials and methods

### Plant materials and growth conditions

The purslane seeds were sown in 10 cm diameter and 10 cm deep plastic pots with vermiculite after sterilization at 4°C in the dark for 3 d. Plants were grown in growth room with temperature controlled at 28 ± 1°C, 65–75% relative humidity, and 16 h light and 8 h dark cycle. The plastic pots were rotated daily to minimize the influence of environments. To determine individual drought, heat and combined drought and heat stress tolerances, 21-d-old seedlings were subjected to three treatments as follows. Drought: no water irrigation; heat: water applied regularly but subjected to 42°C in the incubator; the combination of drought and heat: no water irrigation and subject to 42°C in the incubator. The control treatment was irrigated regularly under 28°C. Based on preliminary experiments, samples were harvested after the treatment for 7 days. A part of samples were determined some physiological indexes directly and the other samples were frozen in liquid nitrogen and stored at −80°C for the other physiological and metabolic parameter analyses. Experiments were executed in triplicates.

### Electrolyte leakage assay and measurement of MDA

EL was determined as described by Shi et al. (2012) with slight modifications using conductivity meter (Leici-DDS-307A, Shanghai, China). Briefly, five intact leaves were incubated in 20 mL deionized water, and shaken for 6 h at room temperature. The initial conductivity (Ci) was measured. Then samples were boiled for 20 min to induce all electrolytes completely. Until cooling to room temperature, the conductivity (Cmax) was determined. Relative EL (%) = (Ci/Cmax) × 100. Malonaldehyde (MDA) was extracted with the trichloroacetic acid (TBA) as described previously (Shi et al., 2012, 2015) and determined at 450, 532, and 600 nm wavelength using Multiskan MK3 (Thermo Scientific, Waltham, MA, USA).

### Determination of soil water content, leaf water loss, and leaf water content

To assess soil water status, SWC was detected using soil moisture temperature recorder (Fotel Precise Instrument Company, Shanghai, China) at harvest time. To determine purslane drought related stress tolerance, LWL was assayed. The detached leaves from the different treatments were placed on the weighting paper respectively at the same room were quantified every 1 h intervals for successive 8 h. the leaf water loss was calculated from the decrease in the rate of FW at specific time intervals. For measurement of LWC, leaf samples were harvested after treatment. Fresh weight (FW) was measured after harvest immediately, and the dry weight (DW) was measured after 16 h incubation at 80°C oven. LWC (%) was calculated as (FW−DW)/FW × 100%.

### Determination of ${\text{O}}_{2}^{•-}$, antioxidant enzyme activities and chlorophyll content

${\text{O}}_{2}^{•-}$ content was measured by the SOA Assay Kit (Elisa, Shanghai, China). Based on the antibody-antigen reaction, ${\text{O}}_{2}^{•-}$ content was determined by measuring the absorbance of TMB substrate at 450 nm.

Superoxide dismutase (SOD) activity was assayed with the SOD Assay Kit (Beyotime, Shanghai, China). 2-(4-iodophenyl)-3-(4-nitrophenyl)-5-(2, 4-disulfophenyl)-2H-tetrazolium (WST-1) was used in this assay, which couples with xanthine oxidase (XO) to generate ${\text{O}}_{2}^{•-}$ and formazan dye. This reaction is inhibited by SOD which catalyzing ${\text{O}}_{2}^{•-}$ into O_2_ and H_2_O_2_. Therefore, SOD activity can be measured by the absorbance of formazan dye at 450 nm.

To determine the POD activity, Plant POD activity Assay Kit (Jiancheng, Nanjing, China) was used. Based on the guaiacol oxidation, POD activity was calculated by measuring the absorbance of reaction buffer at 340 nm.

The CAT activity was measured by CAT Assay Kit (Beyotime, Shanghai, China). Briefly, the protein supernatants were treated with excess H2O2 for decomposition by CAT, and the remaining H_2_O_2_ coupled with a substrate was treated with POD to generate a red production, which can be determining the decomposition of H_2_O_2_.

Chlorophyll content was determined as previously described with slight change (Frank et al., 2005). Briefly, leaf samples were treated at 95°C for 30 min. After that, the leaf samples were dried to constant weight at 80°C. Then the samples were thoroughly homogenized and chlorophyll was totally extracted with 96% (v/v) ethanol in the dark at 4°C. For chlorophyll quantification, the supernatant of ethanol extract was measured at 649 nm and 665 nm wavelengths.

### Metabolite analysis by GC-MS (gas chromatography-mass spectrometry)

Lyophilized leaves were extracted with 1,400 μL of 100% methanol (pre-cooled at −20°C). 60 μL ribitol (0.2 mg/mL) was used as an internal quantitative standard and samples were vortexed and centrifuged. After that, the 750 μL chloroform (−20°C) and 1500 μL deionized water were added to the collected supernatant. After centrifugation, 150 μL of the polar phase was dried under vacuum for derivatization. The samples were derivatized using methoxyamination and methyl-trimethyl-silyl-trifluoroacetamide (MSTFA) as described (Lisec et al., 2006).

The metabolites were determined using GC-TOF-MS (Agilent 7890A/5975C, USA). For GC-TOF-MS, 1 μL of derivatizated extract was injected into a DB-5 MS capillary (30 m × 0.25 mm inner diameter × 0.25 μm film, Agilent J & W GC columns, USA). The inlet temperature was set at 260°C. After a solvent delay for 6 min, initial GC oven temperature was set at 60°C; after injection for 1 min, the GC oven temperature was raised to 280°C for 15 min. The injection temperature was set to 280°C and ion source temperature was matched. Helium was used as the carrier gas with a constant flow at 1 mL per minute. The measurement was performed with electron impact ionization (70 eV) in the full scan mode (*m/z* from 30 to 550). The total ion chromatogram (TIC) and mass spectra were evaluated using the program Chem Station (Agilent) and detected peaks were identified by comparison with authentic standards and the database of NIST Spectral Library (NIST 2005, WILEY 7.0).

### Statistic analysis

Data shown are mean ± stand deviation (SD). Statistical analysis was performed using Duncan's multiple range tests (*P* < 0.05). Different letters above the columns in the figures indicated significant differences in relative to control.

## Results

### Effect of stress treatments on plant growth, SWC, LWC, and LWL

**Figure 1:**
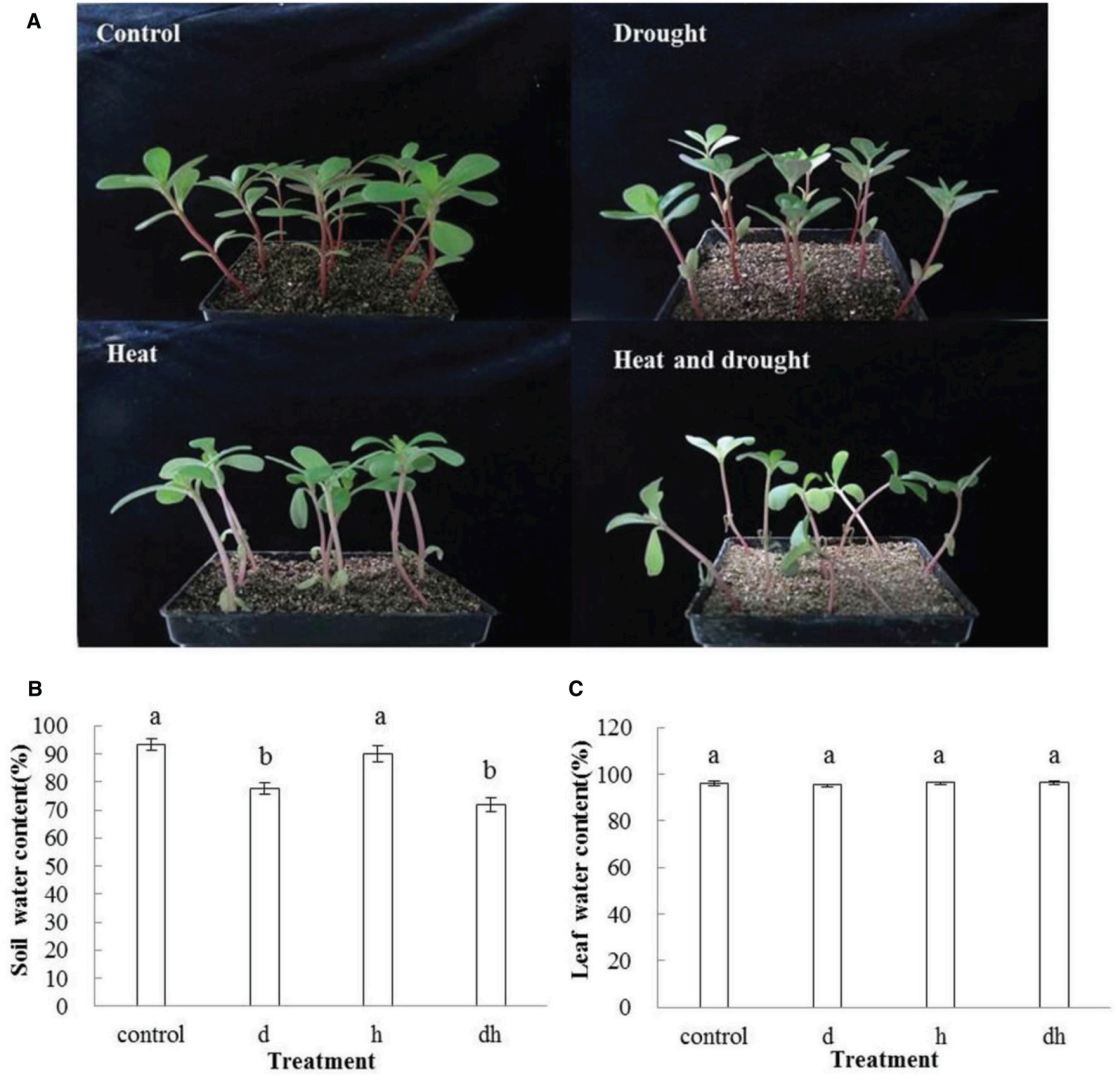
**Effect of drought, heat and combined stress on growth of purslane and water content. (A)** The representative picture of purslane under different treatments; **(B)** Soil water content; **(C)** Leaf water content. Soil water content was measured from 5 pots and leaf water content was measured from 10 leaves. The experiment was repeated three times. d: drought treatment; h: heat treatment; dh: drought combined heat treatment.

**Figure 2:**
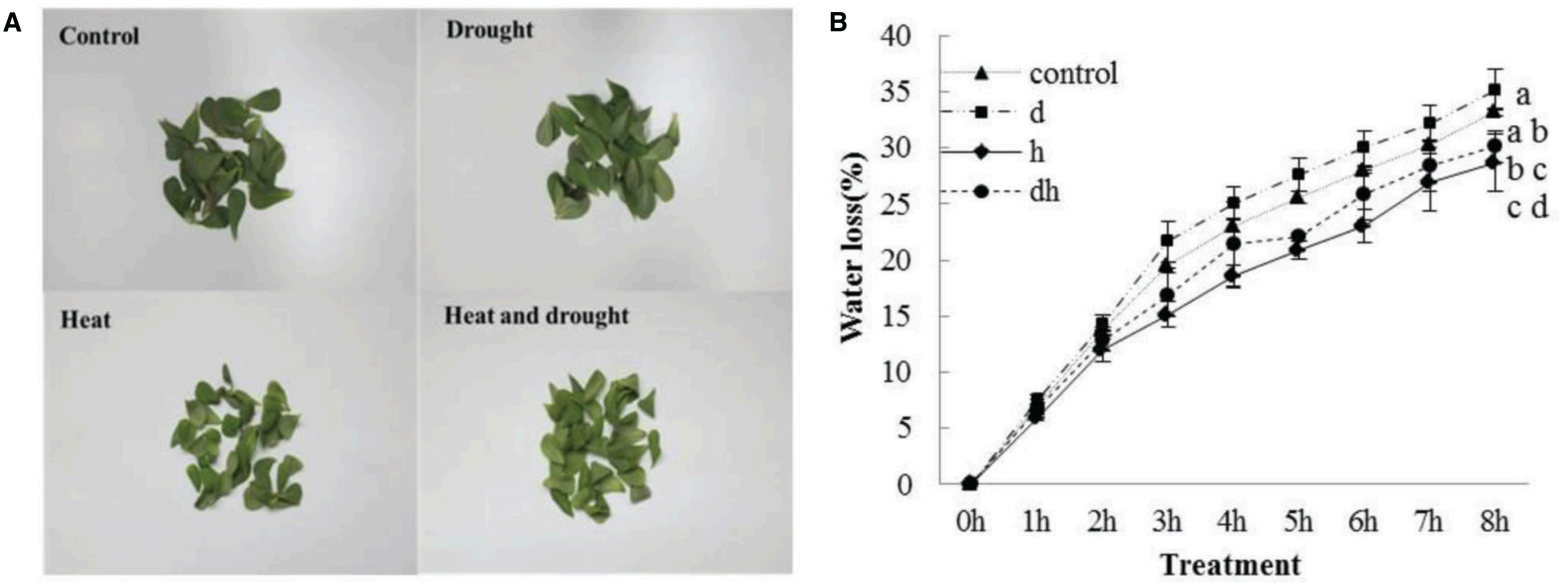
**Leaf water loss in purslane leaves under drought, heat and combined stress condition. (A)** The representative picture of purslane leaves from different treatments detached for 8 h; **(B)** leaf water loss (% change in leaf FW). The leaf tissues were collected from a minimum of 10 independent plants and the experiment was repeated three times. d: drought treatment; h: heat treatment; dh: drought combined heat treatment.

After drought, heat and combined stress treatments, the purslane plants showed retarded growth. The leaf showed decreased fleshy and reduced leaf area, and the stem color was reddish after three treatments. Under heat and combined stress condition, the purslane slouched obviously, and the cotyledons were completely wilting (Figure 1A). Moreover, SWC dropped significantly to 78 and 72% under drought and combined stress conditions, respectively, compared with that under the control (93%) and heat stress (90%) conditions (Figure 1B). As succulent plant, LWC of purslane in control condition was as high as 96% and only declined to 95% after drought treatment. There were no significant differences between the control and stress treatments (Figure 1C). After leaf cut, LWL reached 25–35% under different conditions. LWL under heat and combined stress were lower than that in the control and drought stress (Figures 2A,B).

### Effects of different treatments on purslane MDA and EL

**Figure 3:**
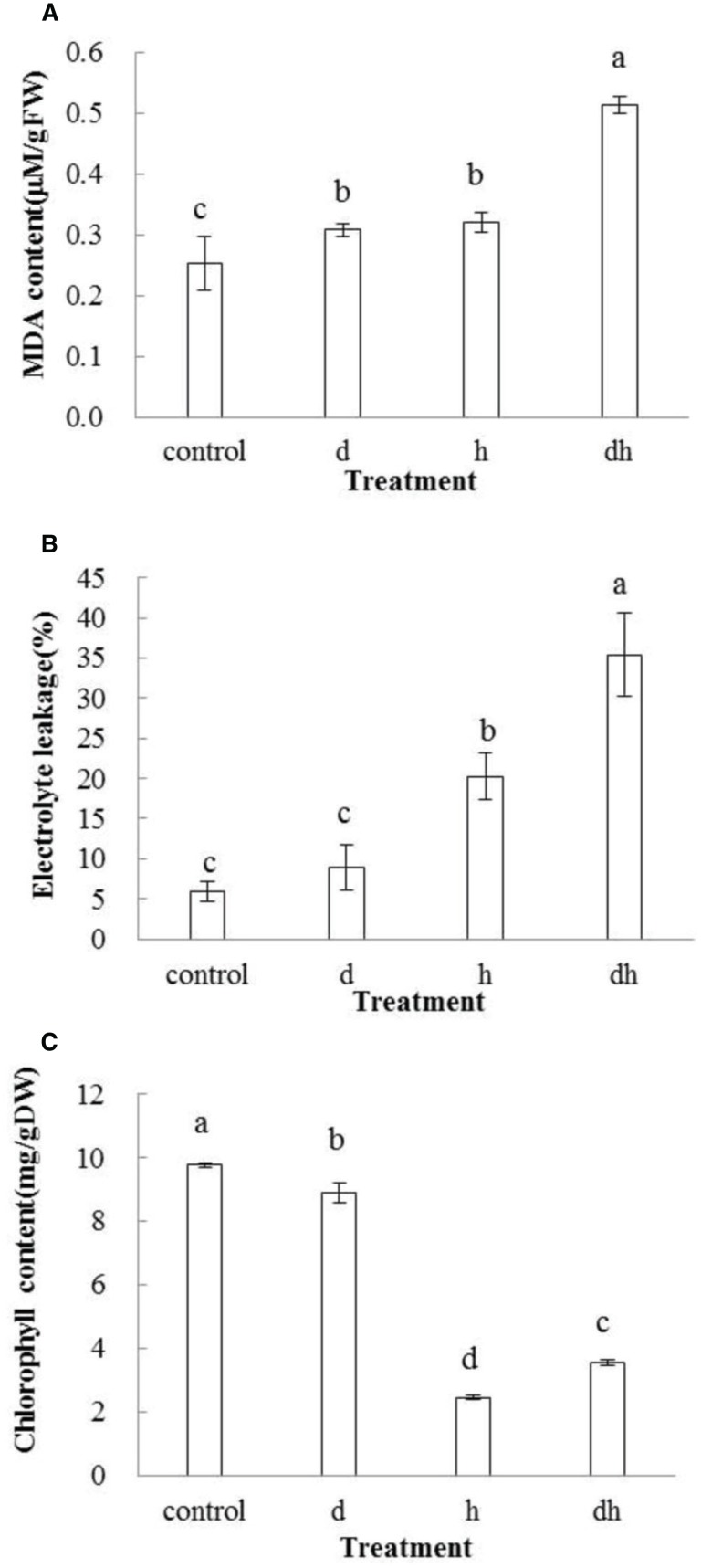
**Changes of MDA, EL, and chlorophyll contents in purslane drought, heat and combined stress. (A)** MDA content; **(B)** EL; **(C)** Chlorophyll content. The results shown are mean means ± standard deviation (SD). All data were collected from 10 seedlings and the experiment was repeated three times. d: drought treatment; h: heat treatment; dh: drought combined heat treatment.

Malondialdehyde content increased after stress treatments in purslane. In drought or heat stressed plants, MDA content was significantly higher than that in control, while plants subjected to combined stress exhibited highest MDA content (Figure 3A). EL showed the same tendency as MDA after stress treatments, and significant differences were observed after heat stress as well as combined stress treatments (Figure 3B). These results indicated that combined stress caused more serious damage to purslane than individual stress.

### Effect of different treatments on purslane chlorophyll content

After drought treatment, chlorophyll content in purslane decreased slightly. However, individual heat and combined stress treatments caused significant reduction than drought and the control (*P* < 0.05), indicating that heat was the primary effect inhibiting the chlorophyll biosynthesis (Figure 3C).

### Effect of different treatments on purslane ROS and antioxidant enzyme activities

**Figure 4:**
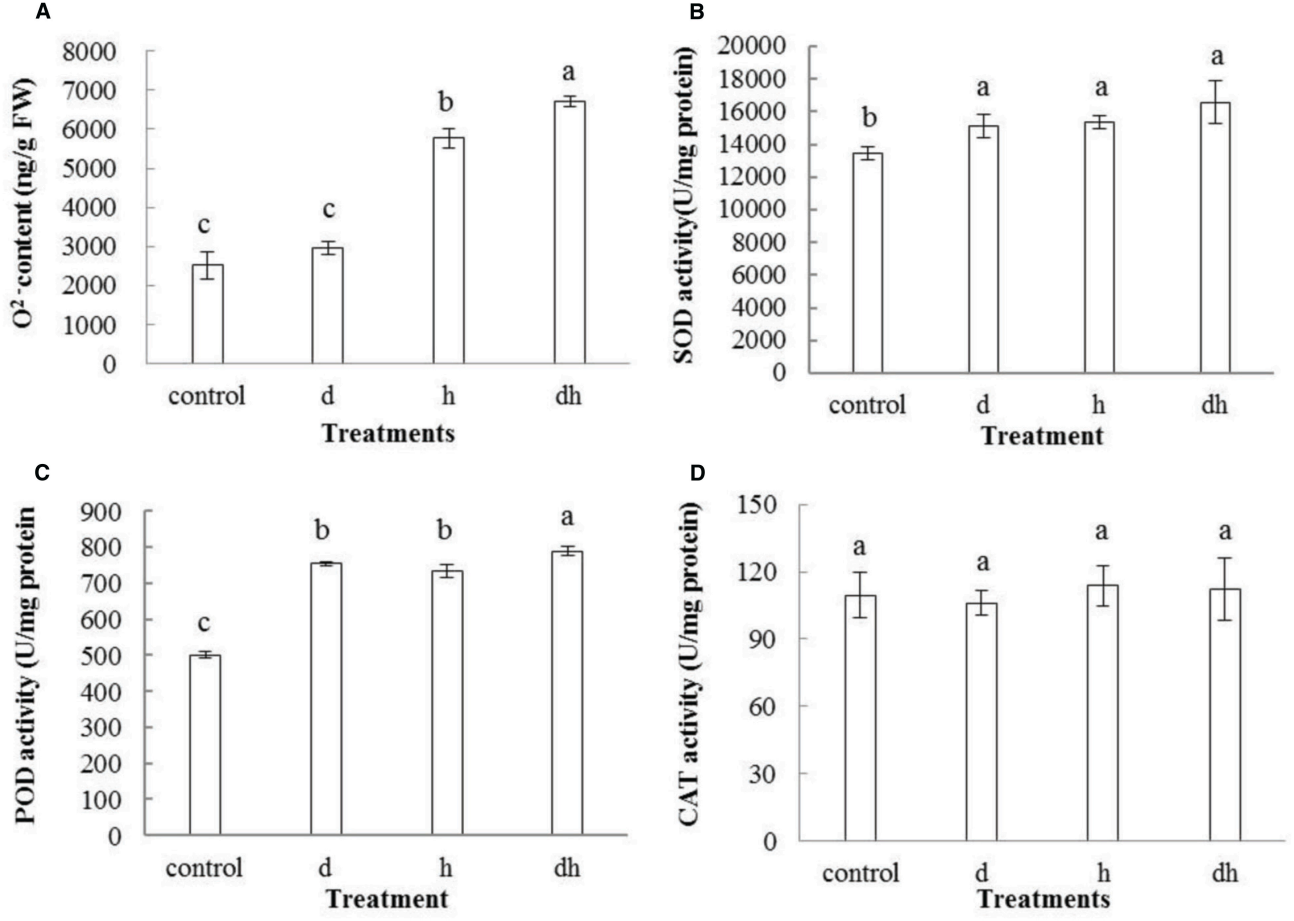
**Effect of drought, heat and combined stress on ROS metabolism in purslane. (A)** ${\text{O}}_{2}^{•-}$ content; **(B)** SOD; **(C)** POD; and **(D)** CAT activity. The results shown are mean means**i**standard deviation (SD). All data were collected from 10 seedlings and the experiment was repeated three times. d: drought treatment; h: heat treatment; dh: drought combined heat treatment.

Various environmental stresses usually caused increasing generation of ROS. In line with the previous studies (Shi et al., 2012, 2015), ${\text{O}}_{2}^{•-}$ content also increased after stress treatments. Combined stress treatment resulted in highest ${\text{O}}_{2}^{•-}$ content in purslane plants (Figure 4A). POD, SOD and CAT play vital roles to detoxify ROS over-production in plants. Activities of SOD and POD increased significantly in purslane after three treatments (Figures 4B,C), while CAT activity showed no significant change under stressed conditions (Figure 4D).

### Metabolic profiling of purslane leaves under different conditions

Totally 37 metabolic compounds were detected in purslane, including 15 amino acids, 7 organic acids, 11 sugars, 3 sugar alcohols and urea. Fructose, galactose and xylitol were only detected in the control plant indicating that these compounds degraded after different stress treatments. Alanine, sorbose, glucose, and heptulose were only detected under drought treatment, while glycine, threonine, and asparagine were detected after heat treatment. In addition, we detected isoleucine and phenylalanine only in the combined stress treated plants. Propionic acid, gluconic acid, mannose, and urea were detected in all treatments and induced by both individual and combined stresses (Table 1).

**Table 1:** **The metabolites contents (data shown are the mean) under different treatments and ratios compared with the control**.

		**Absolute content (μg/g)**				**Relative content to control**		
**Metabolites**	**Name**	**Control**	**Drought**	**Heat**	**Combined**	**Drought**	**Heat**	**Combined**
Amino acid	Valine	3.71	–	9.92	5.08	**D**	2.67	1.37
	Serine	4.04	9.74	–	–	2.41	**D**	**D**
	Proline	24.09	25.88	25.06	13.55	1.07	1.04	**0.56**
	Aspartic acid	4.84	–	–	2.93	**D**	**D**	**0.61**
	Glutamic acid	4.1	7.56	3.63	–	1.84	**0.88**	**D**
	Ornithine	1.99	–	19.94	8.61	**D**	10.03	4.33
	Alanine	–	3.31	–	–	**I**	/	/
	Glutamine	–	–	23.54	9.43	/	**I**	**I**
	Tyrosine	–	–	4.38	3	/	**I**	**I**
	Tryptophan	–	–	4.3	3.25	/	**I**	**I**
	Glycine	–	–	3.1	–	/	**I**	/
	threonine	–	–	1.51	–	/	**I**	/
	Asparagine	–	–	3.09	–	/	**I**	/
	Isoleucine	–	–	–	9.09	/	/	**I**
	Phenylalanine	–	–	–	5.6	/	/	**I**
Organic acid	Ethanedioic acid	60.34	112.85	102.34	80	1.87	1.7	1.33
	Butanedioic acid	21.99	–	8.42	3.5	**D**	**0.38**	**0.16**
	Benzenedicarboxylic acid	24.16	32.68	21.2	19.89	1.35	**0.88**	**0.82**
	Hexadecanoic acid	45.39	32.89	31.87	27.84	**0.72**	**0.7**	**0.61**
	Octadecanoic acid	45.04	40.78	36.74	40.39	**0.91**	**0.82**	**0.9**
	Propanoic acid	–	3.69	16.11	9.49	**I**	**I**	**I**
	Glucaric acid	–	3.8	5.07	3.03	**I**	**I**	**I**
Sugar	Galactose	2.66	–	–	–	**D**	**D**	**D**
	Turanose	1.97	–	2.55	–	**D**	1.29	**D**
	Allose	5.75	4.46	6.69	6.54	**0.78**	1.16	1.14
	Glucopyranoside	127.66	68.57	44.47	36.66	**0.54**	**0.35**	**0.29**
	Sorbose	–	1.24	–	–	**I**	/	/
	Glucose	–	3.04	–	–	**I**	/	/
	Fructose	1.85	2.74	–	–	**I**	**D**	F
	Mannose	–	5.9	3.22	2.52	**I**	**I**	**I**
	Cellobiose	–	0.78	2.29	–	**I**	**I**	/
	Heptulose	–	1.57	–	–	**I**	/	/
	Galactinol	–	–	9.26	5.33	/	**I**	**I**
Sugar alcohol	Xylitol	2.95	–	–		**D**	**D**	**D**
	Myo-Inositol	6.68	8.19	19.84	14.33	1.23	2.97	2.15
	Arabitol	–	–	12.53	6.53	/	**I**	**I**
Others	Urea	–	12.29	112.74	65.13	**I**	**I**	**I**

D: indicates detected in the control but not in treatments; I: indicates detected in the treatments but not detected in the control; –: indicates not detectable; Color scales are as following:

D    ≤1.0    1~2    2~4    ≥4.0      I

**Figure 5:**
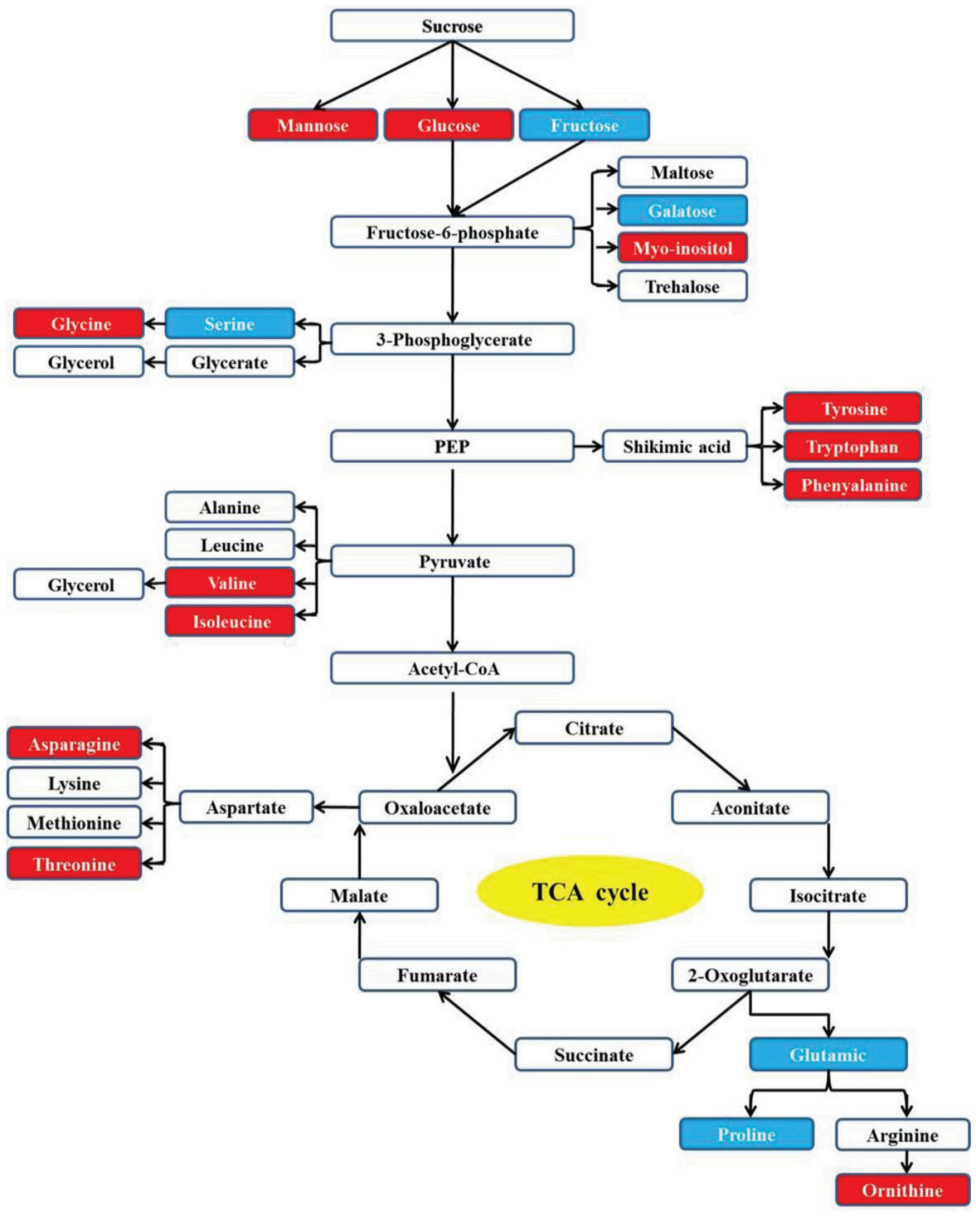
**Carbon and amino acid metabolism pathways changed by drought, heat and combined stress in purslane**. Red shading indicates increase under heat or combined stress; blue shading indicates decrease under heat or combined stress.

Through pathway analysis, 17 metabolites were directly involved in the glycolysis and TCA metabolic pathways. Among them, 12 metabolites increased under the heat or combined stress conditions, while the other 5 metabolites decreased in those conditions. This result further confirmed that the carbon and amino acid metabolisms were extensively modulated by stresses (Figure 5).

## Discussion

Under varying environmental conditions, plants encounter a wide range of abiotic stresses. Plants develop a variety of stress responses that enable them to tolerate adverse conditions and survive. Many studies revealed key signaling transduction pathways for different abiotic stresses, such as ABA pathway for drought, C-Repeat Binding Factor (CBF) pathway for cold, and Salt Overly Sensitive (SOS) pathway for salt (Jaglo-Ottosen et al., 1998; Mittler, 2006). Studying abiotic stress individually is valuable but it can be misleading because plant responses to the combination of abiotic stresses are different from these to individual stress (Chinnusamy et al., 2004; Rizhsky et al., 2004; Atkinson and Urwin, 2012).

In this study, we compared physiological and metabolic changes of purslane plants to drought, heat, and combined drought and heat stresses. The results showed that the combined stress caused more severe physiological damage than drought or heat individual stress as evidenced by increased MDA and EL content (Figures 3A,B). When subjected to various environmental stresses, plant cell membrane plays a significant role in maintaining cell turgor pressure and physiological functions. EL has been widely used as a key parameter to estimate cell membrane stability (Hu et al., 2010; Zhao et al., 2011). MDA is an indicator of lipid peroxidation under adverse environment, and is formed by ROS induced decomposition of polyunsaturated lipids (Pryor and Stanley, 1975). In line with MDA data, combined stress caused greater increase of ${\text{O}}_{2}^{•-}$ content than that in the control or individual stress treated plant (Figure 4A). Antioxidant enzymes such as POD, SOD, and CAT play crucial roles in antioxidant system, which are activated to scavenge the redundant ROS and protect plant cells (Naya et al., 2007). A pronounced increase in POD and SOD activities was observed after drought or heat treatments, and especially after the combined stress treatment (Figures 4B,C). These results indicated that purslane plants suffered greater damage when subjected combined drought and heat stress.

Plants subjected to drought typically exhibit characteristic losses in leaf water content (LWC; Westgate and Boyer, 1985). However, purslane still kept high water content in leaves when exposed to drought (Jin et al., 2015). In this study, we further observed that there was no distinct difference for the LWC in purslane under individual stress or combined stress conditions (Figure 1C). Drought and heat induce abnormal transpirational water loss, which has cooling effect but also causes rapid cell desiccation (Nobel, 1987). Purslane is dicotyledonous and one of the C_4_ plants displaying Kranz anatomy structure, which has a palisade parenchyma in leaf (Ren et al., 2011). C_4_ plants have high water use efficiencies (WUE_*S*_), and the presence of the CO_2_ concentrating mechanisms makes C_4_ photosynthesis more competitive in conditions that promote carbon loss through photorespiration, such as high temperatures, high light intensities, and decreased water availability (Edwards et al., 2004). C_4_ photosynthesis is characterized by the presence of a metabolic CO_2_ pump that concentrates CO_2_ in the vicinity of the main enzyme of carbon dioxide fixation ribulose-1, 5-bisphosphate carboxylase/oxygenase (Edwards et al., 2004). This confers a number of important advantages in terms of WUE due to allowing high rates of photosynthesis to occur even when stomata are closed while limiting flux through the photo-respiratory pathway (Edwards et al., 2001). This special character might explain why purslane had high level of LWC after exposure to drought, heat or combined stress. Additionally, leaf water loss at 8 h after detachment showed no significant difference between the control and drought related stress (Figure 2). Dehydration avoidance mechanisms involve the maintenance of a high (favorable) plant water status during stress (Lopes et al., 2011). Such strategies include minimized water loss (e.g., stomatal closure, reduced leaf area, and senescence of older leaves) or maximal water loss afforded by increased root proliferation at depths where the water is available (Lopes et al., 2011). Therefore, high water reservation and low water loss might be attributed to purslane resistance to stresses.

To cope with adverse environment, plants accumulate osmolytes to alleviate cellular hyperosmolarity and ion disequilibrium. Proline is best-known compatible solute in plant, which fulfills protective role through a range of mechanisms, such as supporting osmotic adjustment, protection of cellular structures, and regulation of cellular redox potential (Hare et al., 1998). Proline was slightly accumulated under individual drought and heat conditions, but not by combined stress. These findings were consistent with the studies which reported combined drought and heat condition (Rizhsky et al., 2004; Prasch and Sonnewald, 2013).

Other metabolites also play key roles during plant stress responses. We observed an increase in the level of myo-inositol and galactinol, which are precursors of the synthetic route of the raffinose family oligosaccharides (RFO) (Albini et al., 1999; Peterbauer and Richter, 2001). RFO has been hypothesized to act on cell stabilization during drying through interacting with the phosphate groups bound to lipids of membranes and with macromolecules, altering the fluidity of cytoplasm in a process characterized by reversible cell vitrification (Farrant et al., 2012). Furthermore, several amino acids including valine, ornithine, glutamine, tyrosine, and tryptophan increased under heat and combined stress conditions. The increases of valine and tyrosine could be an indicative of deposition of extensins in cell wall. Extensins are classified as members of a family of glycoproteins enriched in hydroxyproline, which provide stability to cell wall (Cassab, 1998). Ornithine is the intermediate compound in the arginine biosynthesis where the pathway divaricates to the production of compounds that are known as osmoprotective substances (Kalamaki et al., 2009). The accumulation of these amino acids under heat and combined stress conditions suggested a cellular osmotic adjustment for keeping the leaf turgidity during plant response to these stresses. The accumulation of asparagine in vegetative tissues occurs in response to saline and water stresses (Lea et al., 2007). In present study, the asparagine content increased in purslane under heat condition, which was in agreement with report that asparagine accumulates to a considerable extent under stress conditions (Lea et al., 2007). Accumulation of amino acids can be also associated to storage of available substrate for protein synthesis and quick recover of the plant metabolism after stress (Suguiyama et al., 2014).

On the other hand, the tryptophan, tyrosine and phenylalanine are aromatic amino acids, which locate the downstream of shikimic acid in metabolism pathway. These amino acids increased in purslane under stress conditions suggested a change in metabolism toward the secondary metabolite production, which developed an important role on stress tolerance (Suguiyama et al., 2014).

Urea is a nitrogen-containing metabolite, which is usually related to protein degradation and showed the greatest increase in drought-stressed leaves (D'Andrea et al., 2014). Urea is an osmolyte, but high levels urea disturbs protein folding and binding (Laxson et al., 2011). In plants, urea is synthesized in the mitochondria but accumulated in the vacuole (Witte, 2011). In purslane, high level of urea in the vacuole reduced the negative effects contributing to osmolarity maintenance (D'Andrea et al., 2014).

Additionally, the metabolite profile of plants suffered to combined drought and heat was more similar to that of heat treated plants than to that of the control or drought treated plants. The effect of drought on metabolites was conserved.

This might be due to the factor that purslane is one of the C_4_ plants, which are considered to have mastered the art of drought control particularly as they are able to maintain leaf photosynthesis with closed stomata(Lopes et al., 2011). On the other hand, the metabolic pathways may be regulated to meet the metabolic demands under each stress condition, resulting in additive metabolite profile under combined stress condition (Obata et al., 2015). Moreover, 7-day drought stress is a short term treatment which might explain the limited effect of drought on purslane in this study. Due to innocuous drought stress, similar metabolic changes were found both heat and combined stress. However, metabolic networks are highly dynamic and metabolic profiling only reveals the steady-state level of metabolites, detailed kinetics and flux analyses will be instrumental for a better understanding of metabolic fluctuations in response to stress (Krasensky and Jonak, 2012; Obata and Fernie, 2012).

Taken together, the results in this study indicated that drought, heat, and combined stress severely affect purslane physiological parameters. Combined drought and heat stress caused more serious damage in purslane plant than individual stress. To survive, purslane developed different physiological and metabolic strategies to respond to drought, heat and combined stresses, including increased antioxidants, osmoprotectants and metabolites, and decreased water loss and cell damage.

## Author contributions

RJ and ZC designed the experiment. RJ, YW, RL, and JG performed the experiments and analyzed the data. RJ and ZC wrote the manuscript.

### Conflict of interest statement

The authors declare that the research was conducted in the absence of any commercial or financial relationships that could be construed as a potential conflict of interest.

## Abbreviations

CAT: catalase
EL: electrolyte leakage
GC-MS: gas chromatography-mass spectrometry
LWC: leaf water content
LWL: leaf water loss
MDA: malondialdehyde
${\text{O}}_{2}^{•-}$: superoxide radical
POD: peroxidase
ROS: reactive oxygen species
SOD: superoxide dismutase
SWC: soil water content.
